# High Throughput Discovery and Design of Strong Multicomponent Metallic Solid Solutions

**DOI:** 10.1038/s41598-018-26830-6

**Published:** 2018-06-05

**Authors:** Francisco G. Coury, Kester D. Clarke, Claudio S. Kiminami, Michael J. Kaufman, Amy J. Clarke

**Affiliations:** 1 Center for Advanced Non-Ferrous Structural Alloys, George S. Ansell Department of Metallurgical and Materials Engineering, Colorado School of Mines, Golden, CO 80401 USA; 2 Departamento de Engenharia de Materiais, Universidade Federal de São Carlos, Rodovia Washington Luís, km 235, São Carlos, SP 13565-905 Brazil

## Abstract

High Entropy Alloys (HEAs) are new classes of structural metallic materials that show remarkable property combinations. Yet, often times interesting compositions are still found by trial and error. Here we show an “Effective Atomic Radii for Strength” (EARS) methodology, together with different semi-empirical and first-principle models, can be used to predict the extent of solid solution strengthening to discover and design new HEAs with unprecedented properties. We have designed a Cr_45_Ni_27.5_Co_27.5_ alloy with a yield strength over 50% greater with equivalent ductility than the strongest HEA (Cr_33.3_Ni_33.3_Co_33.3_) from the CrMnFeNiCo family reported to date. We show that values determined by the EARS methodology are more physically representative of multicomponent concentrated solid solutions. Our methodology permits high throughput, property-driven discovery and design of HEAs, enabling the development of future high-performance advanced materials for extreme environments.

## Introduction

Multicomponent equiatomic alloys have garnered considerable interest in the literature over the past decade. One of the main focus areas is to fundamentally understand the high strengths and toughnesses exhibited by some of them^1–3^. Although the properties are extremely promising^4^, the compositional landscape (i.e., the number of possible compositions), is massive, and an almost infinite number of compositions is possible. One of the so-called “core effects”^5,6^ for HEAs suggests that the total compositional complexity (the configurational entropy) correlates directly with strength in single phase alloys. Indeed, several single phase quinary or senary alloys display attractive mechanical properties^7–10^. However, single phase alloys with fewer elements have proven to be stronger than the “parent”, higher entropy alloys that contain 5 elements or more^6^. A notable example is the ternary equiatomic Cr_33.3_Co_33.3_Ni_33.3_ alloy^3,11–13^, which is stronger and tougher than the quinary equiatomic Cr_20_Mn_20_Fe_20_Ni_20_Co_20_ alloy. To date, this Cr_33.3_Ni_33.3_Co_33.3_ alloy is the strongest and toughest face centered cubic (FCC) single phase solid solution from the quinary CrMnFeCoNi system out of hundreds of possible compositions, including several conventional stainless steels. The ternary Cr_33.3_Co_33.3_Ni_33.3_ alloy, which is the benchmark for strength in this work, was discovered in a study that involved producing all possible equiatomic alloys from the CrMnFeCoNi family with an FCC structure^13^. This alloy was effectively discovered by trial and error^13^.

The paradigm of HEA alloy design has shifted significantly since their inception^6^. The concept was mostly based on the aforementioned four “core effects”, where multicomponent, equiatomic HEAs are single phase and exhibit high strengths. Nowadays, these core effects have been challenged^6^. The main interest in HEA alloy design lies in the flexibility of these compositions with respect to phase stability and microstructural design^14^. If high-throughput tools are developed for predicting properties as a function of composition^15,16^, it then becomes possible to perform targeted alloy design, which means designing an alloy with ideal property combinations for a given application. Since there are endless possible alloys to be discovered, it is almost impossible to think there is not a composition best suited for a given application that exists today. Experimental mapping is prohibitive, as surveying the entire composition space would involve the production and characterization of thousands of samples. Finding idealized alloys cannot be a product of luck and extensive trial-and-error experimentation; the compositional landscape is too big for the time and resources that humankind has available. Targeted alloy design must be used to unlock the potential of new HEA compositions and properties for performance in extreme environments.

Although the high-throughput idea is not new for HEAs^6,17^, CALPHAD simulations, for example, are a high-throughput method for predicting phase equilibria that are used in HEA alloy design^6,10,18^. Alloying^19^ and microstructural^20^ evolution have also been linked to strength, but we could not find an example of targeted alloy design for the prediction of HEA solid solution strengthening, the key strengthening mechanism in these alloys, in which a significantly stronger composition was designed based upon *prediction*. Studies exist that show stronger compositions are not necessarily the ones with the highest entropy, and these alloys are also not necessarily equiatomic^21^. Varvenne *et al*. have suggested the strongest compositions should be those with the highest volumetric misfit between the atoms and/or the ones with the highest shear moduli^22,23^. If these ideas are correct, it should be possible to design significantly stronger alloys than existing ones if accurate atomic radii and elastic constants can be determined. By modifying two existing models in the literature with our new EARS methodology, we designed and made a single-phase FCC alloy (from the CrMnFeCoNi family) significantly stronger than the Cr_33.3_Co_33.3_Ni_33.3_ alloy.

Regardless of the mechanism for strain-hardening after yielding, the yield strength determines the baseline strength potential for a given alloy and is controlled by two primary factors: grain size and composition. Refining the grain size always results in higher strengths^20,21^, and can be accomplished via thermomechanical processing (i.e., rolling, forging, etc.). On the other hand, solid solution strengthening is not as straightforward, and is linked to atomic size and elastic moduli mismatch^24^. The former is usually considered the most important. Two general models have been published in the literature to predict the strength of multicomponent solid solutions. One model is proposed by Varvenne *et al*.^22^ for FCC solid solutions, while the other applies to FCC and BCC structures and is proposed by Toda-Caraballo (TC)^25,26^. The Varvenne model is derived from first principles, whereas the second model is semi-empirical and adapts an equation valid for concentrated binary solid solutions. Both models predict composition-yield strength trends, and are thus suitable for high-throughput modeling. The final formulation of these models considers only the size mismatch contribution for strengthening. Therefore, the outcomes of both models are extremely dependent on atomic size input. The elastic constants are also important. The EARS methodology developed here provides accurate and physically significant values of these two properties.

## Results

**Figure 1:**
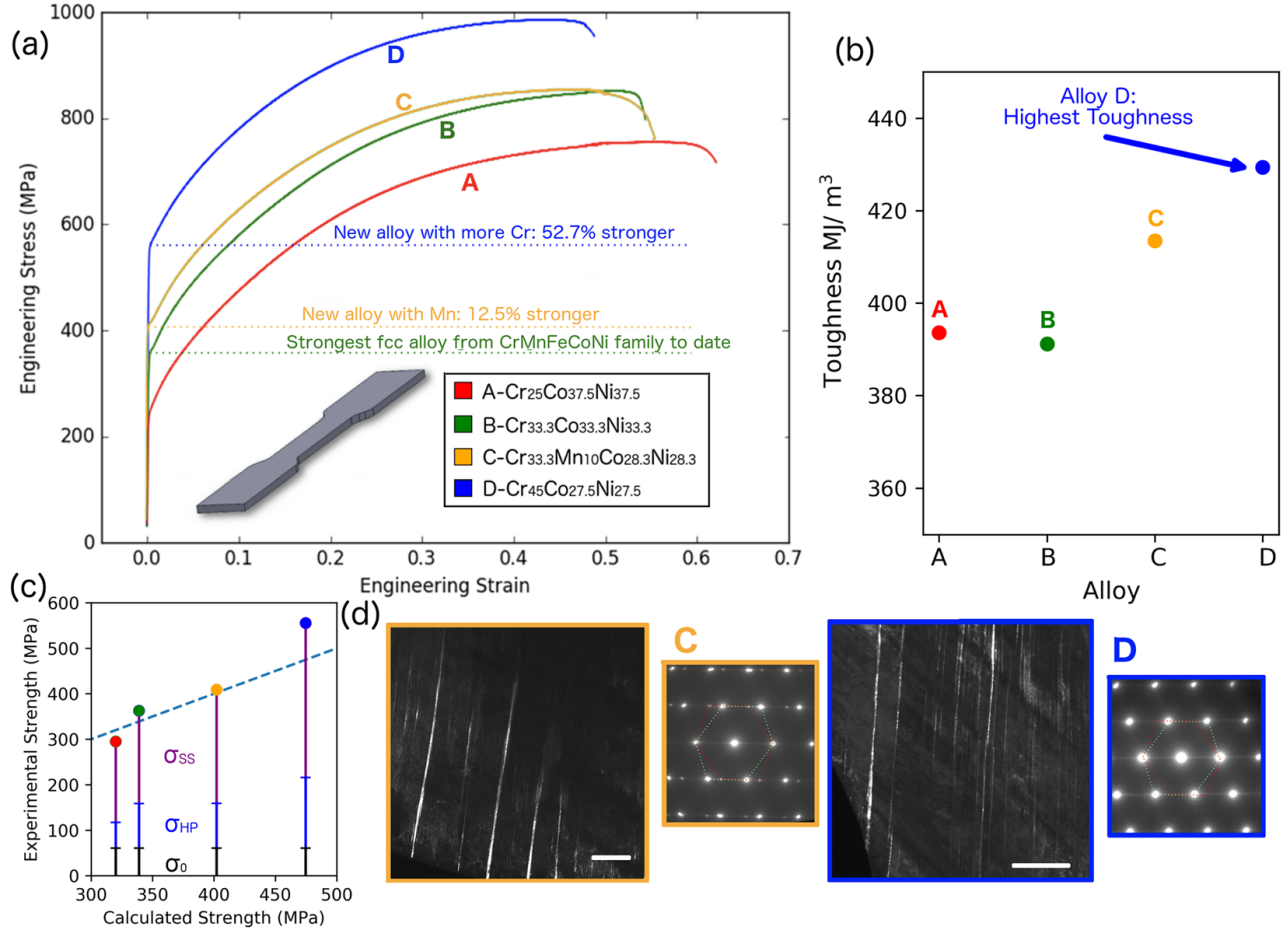
(**a**) Tensile engineering stress - strain curves of Alloys A-D. The Cr_45_Co_27.5_Ni_27.5_ alloy, or Alloy D, is 52.7% stronger than the equiatomic Cr_33.3_Co_33.3_Ni_33.3_ alloy, or Alloy B, regarded as the strongest and toughest FCC solid solution to date from the CrMnFeNiCo family^3,11,13^. Because Alloy D is from the same ternary system as Alloy B, but has lower configurational entropy and is significantly stronger, it is contrary to previous thoughts that strength correlates to entropy. (**b**) The toughnesses extracted from the area under the tensile curves are plotted for the four alloys; Alloy D is the toughest by this criterion. Although fracture toughness tests^15^ may be preferred measurements of toughness, the results shown here demonstrate that Alloy D is indeed tougher than Alloy B. (**c**) Strength predictions with EARS are compared to experimental results. *σ*_*SS*_, *σ*_*HP*_ and *σ*_0_ refer to solid solution strengthening, Hall-Petch strengthening, and base strength (60 MPa for pure Ni), respectively. The determination of these values is described in the Methods section. (**d**) Dark field Transmission Electron Microscopy (TEM) images and selected area diffraction patterns (SADPs) are shown for Alloys C and D after tensile testing, highlighting the presence of fine deformation twins. The dark field images were acquired using the (111) twin reflections; the SADPs were taken from 〈011〉 zone axes for these two alloys. The scale bar in both images is 500 nm.

Strength predictions by EARS and the TC and Varvenne models guided the selection of four experimental HEAs in this work. One alloy (Alloy B) is the ternary equiatomic Cr_0.33_Co_0.33_Ni_0.33_ alloy, which serves as the benchmark for the strongest single-phase FCC alloy reported to date^3,11,27^ from the CrMnFeCoNi family. The reasons for selecting the other alloys will be further discussed below. The tensile stress vs. strain behaviors of the four alloys are shown in Fig. 1. Our EARS methodology predicts the strength to increase from Alloy A to D. With EARS, yield strength of each of the four alloys is predicted. Alloys C and D are 13 and 53% stronger, respectively, than Alloy B. These numbers rise to 25 and 70%, respectively, if we consider only the solid solution strengthening contribution, which can be determined by extracting the other contributions from the total yield strength, as described in the Methods section and shown visually in Fig. 1. Alloy B (the benchmark) has a remarkable combination of strength and toughness^3,11,27,28^, and has been suggested as a potential candidate for cryogenic applications, due to^29^ nanotwinning during deformation. The same nanotwins are observed in Alloys C and D, two of the stronger alloys, as shown in Fig. 1. A simple estimate of toughness, calculated by the area under the stress-strain curve, suggests that the strongest Alloy D has higher toughness than Alloy B, which to our knowledge represents the toughest HEA alloy ever produced (including HEAs outside the CrMnFeCoNi family)^3^. As mentioned previously, this composition was arrived at by *prediction* using the EARS methodology. Although there are several alloys in the literature with higher yield strengths than that of the Cr_45_Co_27.5_Ni_27.5_ alloy designed here, our alloy is a single-phase, coarse grained FCC solid solution, which typically would be expected to have low strength. The absolute yield strength of this alloy can be significantly increased, for instance by grain size reduction, as discussed later. In this work, we attempted to limit this effect. The most impressive property of Alloy D is not its absolute yield strength, but the solid solution strengthening component.

**Figure 2:**
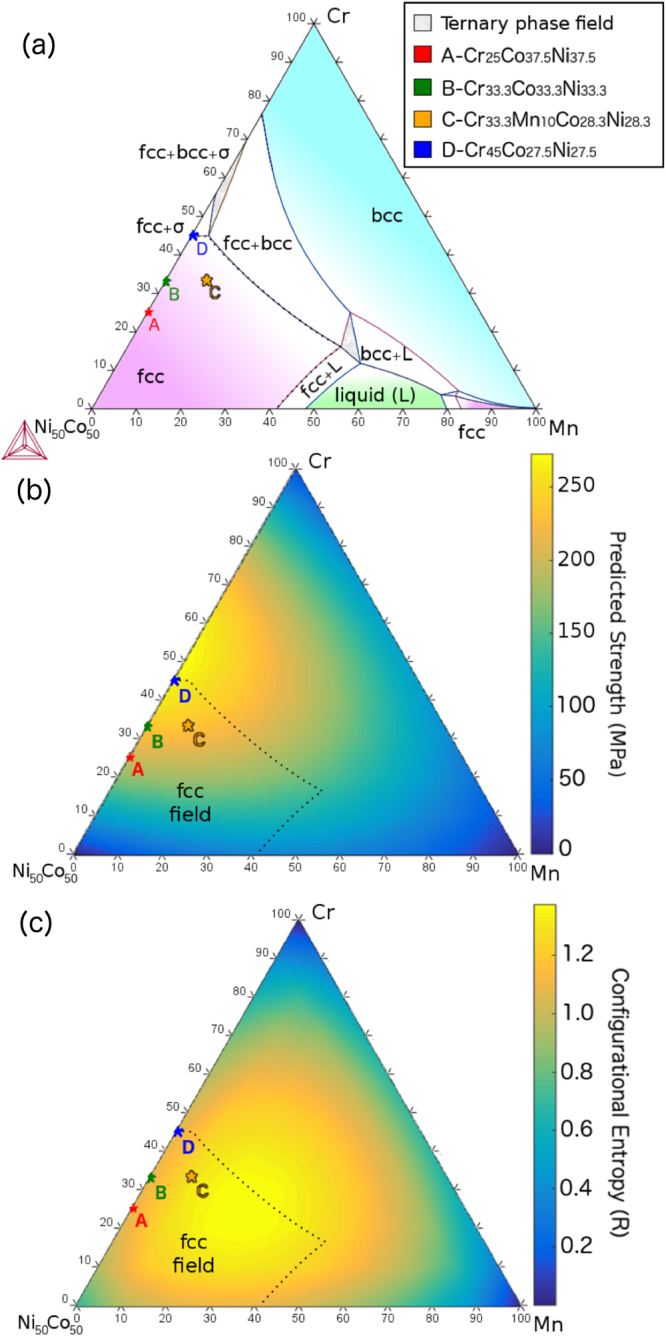
Pseudo-ternary phase diagrams in at% for the Ni_50_Co_50_-Mn-Cr system. (**a**) The 1130 °C isotherm predicted by thermodynamic simulations using the software Thermo-Calc^TM^ and the TCHEA1 database. (**b**) Predicted strengths from the TC model and EARS atomic radii (Table 1). (**c**) Calculated configurational entropy plotted over the same composition space. Clearly, the compositions with maximum configurational entropy do not correspond to those predicted (and shown) to have the maximum strength. The four alloys produced in this study are plotted in all three diagrams. The compositions are given in the legend. The predicted strengths for the allots A-D are 223, 235, 243 and 258 MPa, respectively, considering only the *σ*_*ss*_ contribution.

The predicted strength values using the EARS methodology can be seen in Fig. 2, in which pseudo-ternary phase diagrams are presented. The rationale behind picking Alloys A and D is that they are in the same compositional dimension as Alloy B (Cr_2x_Ni_50−x_Co_50−x_), but with different amounts of lattice distortion due to Cr content (Cr is a larger atom). The EARS methodology predicts increasing strength from Alloys A to D, with Alloy D as the strongest alloy. The fact that subsequent experimentally measured strength increases in order from Alloy A to D strongly supports the methodology used here. The strength calculations also predict that lattice distortion can be increased by adding small amounts of Mn, as shown in Fig. 2, with a resulting increase in yield strength. By adding Mn and removing Ni and Co, the lattice distortion is increased, since we approach a 50% ratio of big and small atoms (Alloy C). This occurs since Mn is bigger than both Co and Ni, but is smaller than Cr (Table 1). Also, Cr is determined to have a larger elastic modulus than Mn in the FCC structure, as shown in the Supplementary Materials section, while Cr-containing alloys have higher shear modulus than Mn-containing ones. On the other hand, Fe is smaller than Mn, so the same effect is observed at reduced magnitude, which explains our selection of Mn over Fe. In the plot of configurational entropy over the same composition space in Fig. 2, it is clear that the composition with the maximum configurational entropy, which is the equiatomic mixture between four elements (Alloy C), deviates completely from that predicted to have the maximum strength using the EARS methodology. This contradicts the original definition of HEAs^6^.

**Table 1:** Atomic radii of the elements used as inputs to the TC model.

Atomic Radii (pm)	Cr	Mn	Fe	Co	Ni
Pure Metal Radii	124.90	130.55	124.12	125.10	124.59
Okamoto Radii	126.90	123.50	121.90	121.90	123.90
Binary Solid Solutions	129.44	130.60	128.81	125.26	124.59
TC-EARS (This work)	129.25	127.52	126.81	124.46	123.28
Varvenne-EARS (This work)	130.09	129.41	127.86	124.30	122.66

The “Pure Metal Radii” were calculated using the lattice constants of the pure metals in their original crystal structures, as given by Pearson’s handbook^36^ for Cr, Fe, Co and Ni. The Mn value is given by Linus Pauling^37^ for the room temperature crystal structure. For both Co and Mn, since the nearest neighbors do not have the exact same distances, the total radii were calculated as half of the weighted average of the nearest neighbor interatomic spacings. The “Okamoto Radii” were calculated by first-principles for CrMnFeCoNi alloys^30^. The “Solutions Radii” were extracted form binary FCC solid solutions by Varvenne *et al*.^22,23^. The EARS radii, or “Effective Atomic Radii for Strength” were calculated in this work. The fact that the TC-EARS values differ from the Varvenne-EARS values is expected, since the setup of each model is different. Using either models yields with EARS provides better strength predictions than any of the other atomic radii sets.

**Figure 3:**
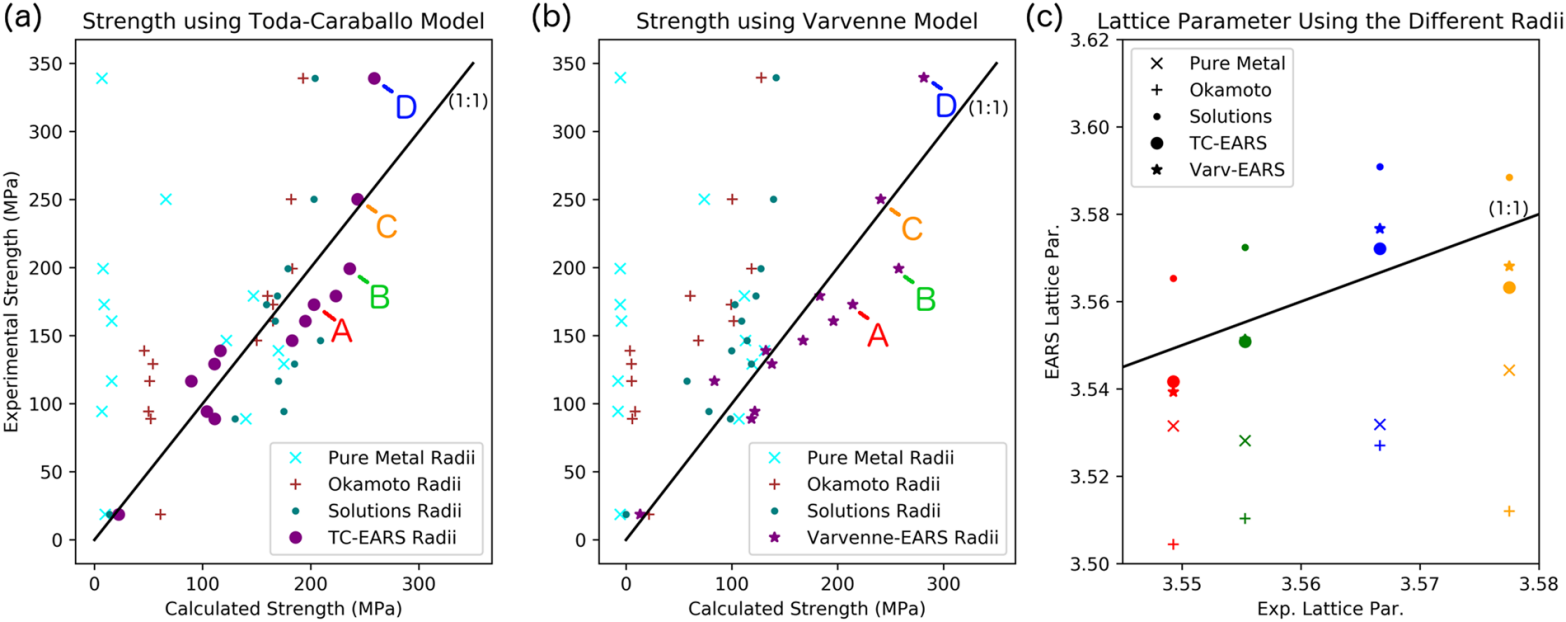
Comparison between the experimental and predicted yield strengths for the four alloys studied in this work using (**a**) the Toda-Caraballo Method and (**b**) the Varvenne method, plus additional alloys from the literature^13^, determined with three different sets of atomic radii (Table 1). The prediction using the “Pure Metal Radii” data is poor, primarily because the size of the Mn atoms is larger than the other atoms. Therefore, all the compositions with Mn are predicted to be strong, whereas the other compositions are predicted to be weak. This is possibly the result of the uncommon crystal structure of Mn at room temperature, which most likely does not represent the atomic bond length that Mn has when it is in an FCC structure with other elements in a concentrated solid solution. Using the “Okamoto Radii”, it is possible to predict the strength vs. composition trends for these alloys; however, the values are generally overpredicted by a factor of 2 or 3. The EARS methodology developed here provides the most accurate strength predictions of the alloys. The alloys from this work are indicated by their respective letter A full table with the compositions plotted in this figure is provided in the Supplementary Materials section. (**c**) The calculated atomic radii of the four alloys produced in this work are compared to experimentally measured lattice parameters determined by XRD (see Methods section). EARS values are not only more accurate in performing strength predictions, but also give the most accurate lattice parameter predictions for HEAs, suggesting EARS values are more physically significant.

The robustness of the EARS methodology is further shown by comparing experimental and predicted strengths for a large number of alloys reported in the literature^13^, calculated with different atomic radii from Table 1. These results are presented in Fig. 3 for both the TC and Varvenne models. The main reason why EARS is so effective in the prediction of the solid solution strengthening is how it is determined. It is not trivial to determine accurate atomic radii for concentrated, multicomponent solid solutions. They can be determined by first principles methodologies, as performed by Okamoto^30^. This atomic radii set is called here the “Okamoto Radii”. They can also be determined by experimentally evaluating the lattice parameters of the pure materials^23^, the “Pure Metals Radii” or by extracting the atomic radii from solid solutions and extrapolating the atomic size of the pure components, the “Solutions Radii”^17,18^. The atomic radii calculated from first principles, or the “Okamoto Radii” and the “Solutions Radii” are more accurate in calculating the experimentally observed strengths of these alloys, but the strength values can still be overpredicted by as much as 100%. The EARS methodology predicts the strength of all the experimental alloys with the least error, for both the TC and Varvenne models. The EARS methodology significantly increases the accuracy of the predictions for all of the alloys examined, including our experimental alloys and those from the literature.

**Figure 4:**
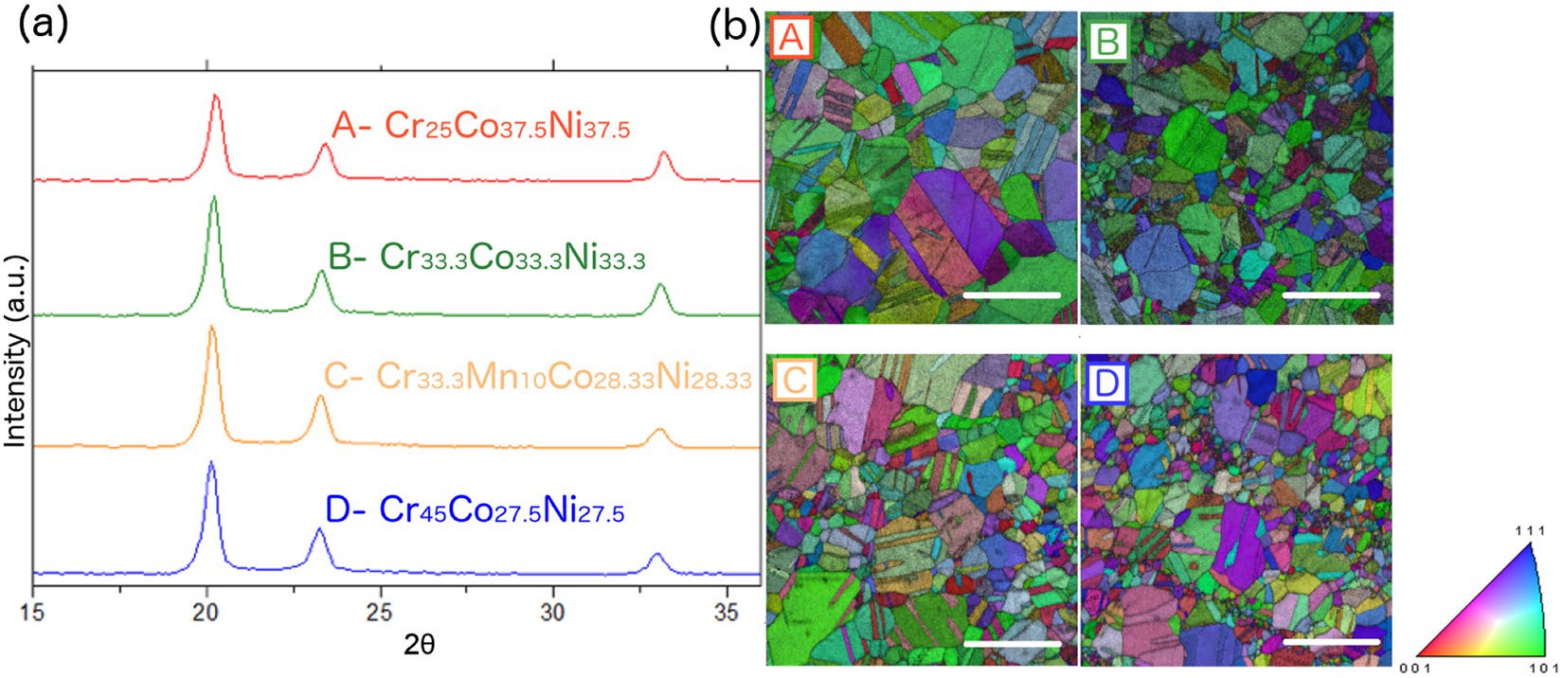
Characterization of as-tested Alloys A-D. (**a**) XRD patterns of the four alloys indicates that each is single-phase FCC solid solution. (**b**) Electron back scattered diffraction (EBSD) inverse pole figure maps are provided, along with an orientation color legend. As can be seen, Alloy A has a slightly more uniform grain size distribution than the other alloys, with an average grain size of approximately 20 µm; the other three alloys have a similar, but slightly wider, distribution of grain sizes, centered around 10 µm. The scale bar corresponds to 100 µm.

Efforts were made in this work to assure that all of the compositions were single-phase solid solutions with equivalent grain sizes. As noted previously, grain size may significantly impact strengthening. The four alloys were cast, rolled and annealed, as described in the Methods section. To ensure the FCC single-phase nature of the final microstructures after annealing, the alloys were investigated by XRD and electron backscattered diffraction (EBSD) as shown in Fig. 4 and, for Alloys C and D, transmission electron microscopy (TEM), as shown in Fig. 1 and also in the Supplementary Materials (Fig. S1-2). These analyses confirmed a single-phase solid solution for all four alloys presented here, and that Alloys B, C and D have similar grain sizes. The Hall-Petch (grain size) influence on the strength of these alloys was subtracted from the strength predictions, as described in the Methods section. This is paramount for the accuracy of the predictions, since the TC and the Varvenne models both predict the solid solution strengthening component.

## Discussion

Considering the CrMnFeCoNi family, only Ni is FCC at room temperature. The exact atomic size changes based upon the crystal structure and the elements in solution. For this reason, the “Pure Metals Radii” will be inaccurate in a solid solution, which will compromise strength model predictions based on lattice distortion. Radii predicted by Density Functional Theory (DFT), or the “Okamoto Radii”, will also have a reasonable degree of uncertainty, due to various factors such as the accuracy of the database, temperature effects and magnetism. The “Solutions Radii” is potentially the most accurate, but reasonable error still exists when predicting the radii of multicomponent solutions, as will be shown. For the EARS method, instead of directly inputting the atomic radii into the TC and Varvenne models, available strength values from the literature were used to calculate the best atomic radii values that match the experimental data. We assume the main factor for solid solution strengthening is indeed atomic size mismatch and the models considered here are accurate. The output are the EARS values, but the EARS methodology also includes the grain size contribution and the elastic moduli of all compositions (see Methods section).

One major concern with the EARS values is that, since they are not experimentally measured and the purpose is to better represent the strength of these alloys, they may have lost their physical significance. This is addressed in Fig. 3. The lattice parameters calculated by the EARS radii are closer to the carefully measured experimental lattice parameters by X-ray (XRD) diffraction of the 4 alloys produced in this work. The lattice parameters determined by EARS are even more accurate than the Solutions Radii, showing that the EARS values are not only more appropriate for the prediction of strength, but are also more physically significant for representing atomic sizes in concentrated multicomponent alloys. This also suggests that EARS radii might also be derived from carefully measured lattice parameters obtained from multicomponent alloys, instead of from strength values as was done here, although we believe that further understanding of short range order in concentrated solid solutions may be necessary. The caveat is that experimental lattice parameter determinations must be performed with care to achieve a high degree of accuracy, since very small changes in an atomic radii set will significantly change the strength predictions.

EARS of HEAs is a powerful methodology that *predicts* lattice strain and solid-solution strengthening. It was shown that the EARS methodology was applied in conjunction with thermodynamic and strength models to improve compositions and strength predictions. The atomic radii are also more physically significant for concentrated solid solutions. Here we apply these ideas for the first time to perform targeted alloy design in the multicomponent landscape, with the goal of maximizing solid solution strengthening. The resulting alloy was 52% stronger than the strongest multicomponent alloy measured from the CrMnFeCoNi family, or 75% stronger if only the solid solution strengthening contribution is considered. This work represents a first step toward property-driven alloy discovery and design of multicomponent alloys. Given the framework of the methodology, it is also possible to include additional property predictions in the future to target optimal compositions for a given application (e.g. strength plus oxidation resistance). The results from this work highlight a new opportunity for the development of strong alloys with different combinations of properties, driving us closer toward high-throughput, property-driven alloy design needed to realize the full potential of HEAs for enhanced performance in extreme environments.

## Methods

The four alloys were prepared by non-consumable arc melting from pure elemental components (99.2% Cr, 99.9% Fe, 99.9% Mn, 99.9% Co and 99.9% Ni). The buttons were flipped and remelted four times to ensure chemical homogeneity; the weight loss of each ingot was determined by making measurements before and after preparation, and was always less than 0.5%. Two buttons from each composition were cross rolled to a thickness of 2.5 mm (~70% cold work), annealed at 1130 °C for 2 h, and then cold-rolled an additional 40% to approximately 1.5 mm. Tensile samples were cut from the resulting plates by electro-discharge machining (EDM), annealed at 1130 °C for 30 min and then water quenched.

X-Ray Diffraction (XRD) was performed with a PanAnalytical Empyrean diffractometer using Mo K_α_ radiation for background signal minimization. Electron Backscattered Diffraction (EBSD) data was collected with a FEI Helios FESEM using an accelerating voltage of 30 kV; the samples were prepared by grinding through 1200 grit SiC papers, polishing sequentially with 6, 3 and 1 µm diamond suspensions for 5 minutes each, and then polishing for 4 h with a Vibromet vibratory polisher and 0.05 µm colloidal silica. The diffraction patterns and inverse pole figure maps are presented as raw data, without any refinement or cleaning performed. For the lattice parameter determination, a Si standard was run at the exact same height as the samples to correct for potential systematic errors. Lattice parameters were calculated using the software HighScore Pro. The deviation of the Si lattice parameter was used to baseline the measured lattice parameters of all the experimental alloys produced in this work.

For TEM analysis, samples were taken from the uniform elongation region of the gauge length of the tensile samples after deformation. They were prepared by grinding to 100 µm and then electropolishing with a 10% sulfuric acid in methanol solution at 30 V and around −20 °C using a Fischione dual jet instrument. The annealed Alloy B sample shown in Fig. S1-2 in the Supplementary Materials was prepared by Focused Ion Beam (FIB) with the FEI Helios and a final cleanup step at 2 kV. The TEM was performed using a FEI Talos microscope operating at 200 kV.

A standard sub-size ASTM E8 tensile sample geometry was used. Tensile tests were performed at a strain rate of 10^−3^ s^−1^, up to failure with a 25.4 mm (1-inch) gage, 50% extensometer. A total of two samples were extracted from each plate of each alloy, resulting in a total of 4 samples per alloy. All the tensile curves are shown in Fig. S1-3 in the Supplementary Materials.

The thermodynamic simulations were performed using Thermo-Calc^TM^ software with the TCHEA1 and Gheno^31^ (Fig. S1-1 in the Supplementary Materials) databases.

The Toda-Caraballo model (TC) was used to calculate the strength and lattice distortion. The first calculation necessary to implement the model is the lattice parameter (a) of the alloy. It can be calculated using the following equation (1).

1 $$a=\frac{1}{f}({x}_{1},\ldots ,{x}_{n})(\begin{array}{ccc}{s}_{11} & \cdots  & {s}_{1n}\\ \vdots  & \ddots  & \vdots \\ {s}_{n1} & \cdots  & {s}_{nn}\end{array})(\begin{array}{c}{x}_{1}\\ \ldots \\ {x}_{n}\end{array})$$

where f is the packing factor of the crystal structure, x_i_ is the atomic fraction of element i, and s_ij_ is the interatomic spacing between the atom i and atom j. This interatomic spacing is estimated by equation (2).

2 $${s}_{ij}=\frac{{(2{r}_{i})}^{2}{K}_{i}{x}_{i}+{(2{r}_{j})}^{2}{K}_{j}{x}_{j}}{2{r}_{i}{K}_{i}{x}_{i}+2{r}_{j}{K}_{j}{x}_{j}}$$

where r_i_ is the atomic radii of atom i and K_i_ is the bulk modulus. The strength of the alloy is estimated as given by equation (3).

3 $$\sigma ={\sigma }_{0}+M\,Z\mu {(\frac{\xi \alpha }{a})}^{\frac{4}{3}}{\delta }^{\ast }$$

The *σ*_*o*_ term is a base strength for the material and was considered to be 60 MPa based upon literature values for pure Ni^13^ and M is the Taylor factor. A value of 3 was used here^25,26^. Z is a fitting parameter used by TC, and here a value of 5 was used. This value was proposed by Toda-Caraballo after fitting experimental data with this model^25,26^. The term *μ* is the average shear modulus; the determination of these elastic constants are explained later, *ξ* is related to the activated slip systems in the crystal (a value of 1 was used here following TC), and *α* is a parameter that accounts for the nature of the dislocations. The TC model uses 16 to account for a mixture of edge and screw dislocations. The δ^*^ is the estimated lattice distortion, and can be calculated by equation (4).

4 $${\delta }^{\ast }=({x}_{1},\ldots ,{x}_{n})(\begin{array}{cccc}0 & {|\frac{da}{{x}_{1}^{2}}|}^{\frac{4}{3}} & \cdots  & {|\frac{da}{{x}_{1}^{n}}|}^{\frac{4}{3}}\\ {|\frac{da}{{x}_{2}^{1}}|}^{\frac{4}{3}} & 0 & \cdots  & {|\frac{da}{{x}_{2}^{n}}|}^{\frac{4}{3}}\\ \vdots  & \cdots  & \ddots  & \vdots \\ {|\frac{da}{{x}_{n}^{1}}|}^{\frac{4}{3}} & {|\frac{da}{{x}_{n}^{2}}|}^{\frac{4}{3}} & \mathrm{...} & 0\end{array})(\begin{array}{c}{x}_{1}\\ \ldots \\ {x}_{n}\end{array})$$

where each term in the matrix estimates the distortion caused by substituting one element from the mixture for another. The simplest way of calculating each of these terms is by considering how much the lattice parameter changes by making a *δx* change in composition. Such as given by equation (5).

5 $$\begin{array}{rcl}\frac{da}{{x}_{1}^{2}} & = & (({x}_{1},{x}_{2},\ldots ,{x}_{n})(\begin{array}{cccc}{s}_{11} & {s}_{12} & \cdots  & {s}_{1n}\\ {s}_{21} & {s}_{22} & \cdots  & {s}_{2n}\\ \vdots  & \cdots  & \ddots  & \vdots \\ {s}_{n1} & {s}_{n2} & \mathrm{...} & {s}_{nn}\end{array})(\begin{array}{c}{x}_{1}\\ {x}_{2}\\ \vdots \\ {x}_{n}\end{array})\\  &  & -\,({x}_{1}-\delta x,{x}_{2}+\delta x,\ldots ,{x}_{n})(\begin{array}{cccc}{s}_{11} & {s}_{12} & \cdots  & {s}_{1n}\\ {s}_{21} & {s}_{22} & \cdots  & {s}_{2n}\\ \vdots  & \cdots  & \ddots  & \vdots \\ {s}_{n1} & {s}_{n2} & \mathrm{...} & {s}_{nn}\end{array})(\begin{array}{c}{x}_{1}-\delta x\\ {x}_{2}+\delta x\\ \vdots \\ {x}_{n}\end{array}))\frac{1}{\delta x}\end{array}$$

The value of *δx* can be a small value. Here, 0.001 was used.

For determining the EARS values, the TC equation was used to calculate the yield strength of all the alloys in Table S1-1. The atomic radii of the elements were varied until the difference of the squares between the calculated strength and experimental strength from the literature^13,32^ were minimized. The “Solutions Radii” were used as a starting point, and the maximum allowable variation of the atomic radii was set to be 3%.

The Varvenne *et al*. model^22,23^ is derived from first principles. In this model, the interaction energy between a dislocation and a solute atom is calculated. The interaction energy is then inputted into a conventional equation to account for thermal aid to overcome the activation energy. The end result is a model for the critical resolved shear stress (converted into yield strength by the Taylor factor) that accounts for strain rate and temperature dependence. The final formulation considers only size misfit contributions, the activation energy for moving a dislocation (Δ*E*_*b*_) and the Peirels Stress at 0 K (*τ*_0_) as follows in equations (6) and (7).

6 $${\tau }_{0}=\,0.051\,{\alpha }^{-\frac{1}{3}}{(\frac{1+\nu }{1-\nu })}^{\frac{4}{3}}\,{f}_{1}({w}_{c}){[\sum _{n}\frac{{x}_{n}\Delta {\bar{V}}_{n}^{2}}{{b}^{6}}]}^{\frac{2}{3}}$$

7 $$\Delta {E}_{b}=\,0.274\,{\alpha }^{\frac{1}{3}}{b}^{3}\,{(\frac{1+\nu }{1-\nu })}^{\frac{2}{3}}\,{f}_{2}({w}_{c}){[\sum _{n}\frac{{x}_{n}\Delta {\bar{V}}_{n}^{2}}{{b}^{6}}]}^{\frac{1}{3}}$$

The *α* term is the proportionality constants between the dislocation line tension and Gb^2^, with G being the shear modulus and b the burgers vector. The value 0.123 was used following Varvenne. The two f(w_c_) functions are called minimized core coefficients, and they account for the non-straight character of the dislocations, that bow out to find local energy minima. The values used were 0.35 and 5.70 for f_1_(w_c_) and f_2_(w_c_) respectively. The most important term is $\Delta {\bar{V}}_{n}$, which is the average volumetric misfit of each atom, calculated as the volume of the n^th^ atom minus the average atomic volume of that mixture. The atomic volume is calculated from the atomic radii given in Table 1. The atomic volume is the volume of the FCC unit cell divided by four, so it is not the atomic volume considering a hard sphere model. The two terms calculated from the equation above are then plugged in the following equation (8) for calculating the yield strength.

8 $${\tau }_{y}(T,\dot{{\epsilon }})={\tau }_{0}\,\exp \,(-\,\frac{1}{0.51}\frac{kT}{\Delta {E}_{b}}\,{ln}\,\frac{{\dot{{\epsilon }}}_{0}}{\dot{{\epsilon }}})$$

In the equation above, k is the Boltzmann constant and ${\dot{{\epsilon }}}_{0}$ is a reference strain rate state set as 10^−4^ s^−1^, again following Varvenne. All the studies performed in this work used a strain rate ($\dot{{\epsilon }}$) of 10^−3^ s^−1^. The calculations were performed to evaluate the strength at 293 K.

For all the elastic constants used as input to both the TC and the Varvenne models, the actual elastic constants measured from ultrasonic techniques reported in the literature were used^13^. For the high-throughput predictions (Fig. 2), the elastic constants were calculated by extrapolating the shear and Poisson moduli to what would be expected from pure single-phase FCC Cr, Mn, Fe and Co. This was performed by fitting a first order polynomial for both moduli values as a function of composition for all of the 10 different alloys used in this work. The final elastic moduli used for a composition were calculated using equations (9) and (10)

9 $${G}_{ss}=103.5\,{x}_{Cr}+81.0\,{x}_{Mn}+51.7\,{x}_{Fe}+81.0\,{x}_{Co}+76.0\,{x}_{Ni}$$

10 $${\nu }_{ss}=0.275\,{x}_{Cr}+0.056\,{x}_{Mn}+0.353\,{x}_{Fe}+0.293\,{x}_{Co}+0.310\,{x}_{Ni}$$

These values were only used for the high-throughput calculations. Note that the elastic constants used for Ni are those from pure FCC Ni. The elastic modulus and bulk modulus were obtained from these two elastic constants using standard conversion equations.

Both the Varvenne and TC models predict only the solid solution strengthening contribution. Therefore, all other strength terms were subtracted from the experimental values. This practice is commonly performed^33^. The total strength of an alloy was considered to be given by equation (11).

11 $$\sigma ={\sigma }_{0}+{\sigma }_{HP}+{\sigma }_{ss}$$

*σ*_0_ is a base strength term explained in the following section, *σ*_*ss*_ is the solid solution strengthening term calculated by the TC and the Varvenne models, and *σ*_*HP*_ is the grain size contribution, accounted for using the Hall-Petch (HP) equation (12).

12 $${\sigma }_{HP}=k{d}^{-1/2}$$

The constant k is material dependent and is usually called the locking parameter; d is the grain size. The k values inputted into the HP equation were extracted from the literature^12,33,34^. Not all the alloys produced in this work had reported locking parameters, so some alloys only had values extracted from hardness tests. The locking parameter seems to escalate with Cr content: Two alloys with Cr–Ni and Ni_60_Co_40_ have a k of 180 MPa µm^−1/2^ ^11^. The quinary Cr_20_Mn_20_Fe_20_Co_20_Ni_20_ has a k of 226 MPa µm^−1/2^ ^34^, the ternary Cr_0.33_Co_0.33_Ni_0.33_ has a k of 265 MPa µm^−1/2^ ^11^, and as shown later, the Cr_0.45_Co_0.275_Ni_0.275_ has a k of 489 MPa *μ*m. This is also true for the HP locking parameters values for the series of alloys measured by hardness, as shown in another study by Wu^35^. The absolute values of the locking parameters extracted by hardness were not used here. The full list of locking parameters used in this work is provided in Table S1-1. All the values were between 180 and 480 MPa µm^−1/2^. For Alloy A, for which 2 different grain sizes were observed, the HP relation estimated from this Cr content matched the strength of the two experimental alloys analyzed. The coarser grain size (around 60 µm) exhibited a yield strength of 255 MPa, while the finer grain size (around 20 *μ*m), had a yield strength of 290 MPa, which match the locking parameter of 250 MPa µm^−1/2^ for the alloys.

Extra attention was given to Alloy D produced in this work. In order to get a more accurate locking parameter for this alloy, a second tensile test on a sample annealed for 2 h instead of 30 min was performed. The experimental stress-strain curve of this material, as well as the microstructure viewed by EBSD, is shown in Figs S1-4 and S1-5 in the Supplementary Materials. This additional sample had a grain size of 80 µm and a tensile strength of 455 MPa, which gives a locking parameter of 480 MPa µm^−1/2^. This is provided in Table S1-1. This value aligns with the higher values of k for samples with higher Cr contents, as this is the sample with the highest k and the highest Cr content.

The experimental strength of pure Ni reported by Wu *et al*.^13^ is about 80 MPa for a grain size of 85 µm. After extracting the HP contribution for this material (k = 180 MPa µm^−1/2^), 60 MPa is still unaccounted for. This is the *σ*_0_ term that accounts for the base strength of Ni at room temperature, although this value is likely slightly higher than what would be expected for a pure single-phase FCC metal. Since all the alloys used in the EARS determination procedure were characterized in the same study by Wu *et al*.^13^, this *σ*_0_ term also accounts for any systematic experimental deviations characteristic of their specific setup.

### Data availability

All data generated or analyzed during this study are included in this published article (and its Supplementary Information files).

## Electronic supplementary material

Supplementary Material
