# Supplementary material for: High Throughput Discovery and Design of Strong Multicomponent Metallic Solid Solutions
Source: Sci Rep. 2018 Jun 5;8:8600. doi: 10.1038/s41598-018-26830-6 (PMC5988724; doi:10.1038/s41598-018-26830-6)
Supplement: Supplementary file 1 — Supplementary Material [file 41598_2018_26830_MOESM1_ESM.pdf]

Title: High Throughput Discovery and Design of Strong Multicomponent Metallic Solid Solutions

Authors: Francisco G. Coury[1], Kester D. Clarke[1], Claudio S. Kiminami[2], Michael J. Kaufman[1], Amy J. Clarke*[1]

Affiliations:

[1]Center for Advanced Non-Ferrous Structural Alloys, George S. Ansell Department of Metallurgical and Materials Engineering, Colorado School of Mines, Golden, CO 80401, USA.

[2]Departamento de Engenharia de Materiais, Universidade Federal de São Carlos, Rodovia Washington Luís, km 235, São Carlos - SP, 13565-905.

*Correspondence to: amyclarke@mines.edu.

Supplementary Materials

Table S1-1. The compositions used to estimate the radii presented in this work and the experimental and calculated strengths. The experimental and solid solution strengthening components are given together with the Locking Parameter used for each composition.

Alloy Composition	*Experimental Strength*	*Locking Parameter*	*Experimental* σ_{ss}	*Reference*	*Toda-Caraballo* σ_{ss}	*Varvenne* σ_{ss}
$Co_{50}Ni_{50}$	110	181	18.7	Wu et al.[8]	22.3	13.3
$Mn_{25}Fe_{25}Co_{25}Ni_{25}$	176	181	88.9	Wu et al.[8]	111.2	88.9
$Fe_{50}Ni_{50}$	186	181	94.3	Wu et al.[8]	103.9	118.6
$Fe_{33.3}Co_{33.3}Ni_{33.3}$	213	181	116.7	Wu et al.[8]	89.6	83.7
$Mn_{33.3}Fe_{33.3}Ni_{33.3}$	222	181	129.2	Wu et al.[8]	110.9	137.8
$Mn_{33.3}Co_{33.3}Ni_{33.3}$	234	181	138.8	Wu et al.[8]	116.3	132.2
$Cr_{20}Mn_{20}Fe_{20}Co_{20}Ni_{20}$	265	226	146.3	Laplanche et al.[33]	182.7	167.3
$Cr_{25}Fe_{25}Co_{25}Ni_{25}$	273	253	160.7	Wu et al.[8]	195.0	196.0
$Cr_{25}Mn_{25}Co_{25}Ni_{25}$	283	253	179.2	Wu et al.[8]	223.2	183.0
$Cr_{25}Co_{37.5}Ni_{37.5}$	295	253	172.7	This work	203.0	214.2
$Cr_{33.3}Co_{33.3}Ni_{33.3}$	363	253	199.3	This work	236.0	257.7
$Cr_{33.3}Mn_{10}Co_{28.33}Ni_{28.33}$	409.2	253	250.1	This work	243.3	240.7
$Cr_{45}Co_{27.5}Ni_{27.5}$	555.4	489	339.5	This work	258.8	281.4

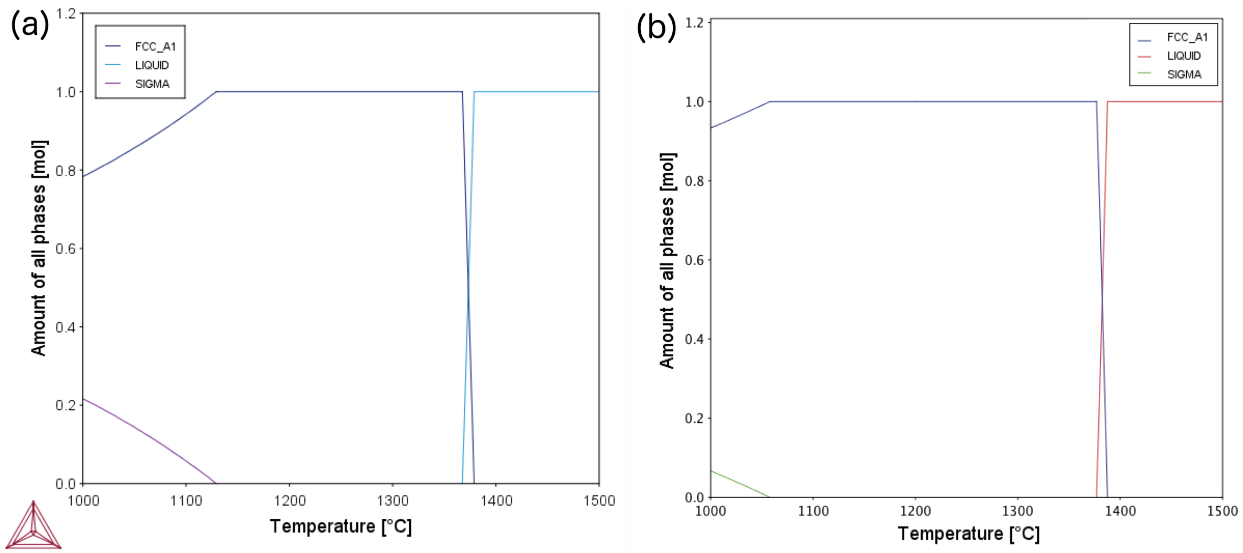

Fig. S1-1. Phase equilibria of the $Cr_{45}Co_{27.5}Ni_{27.5}$ alloy performed using (a) the ThermoCalc^{TM} TCHEA1 database and (b) the Gheno et al. database. Although both predict the alloy to be single-phase at the heat-treating temperature of 1130°C, the Gheno et al. database predicts a larger field for the FCC phase.

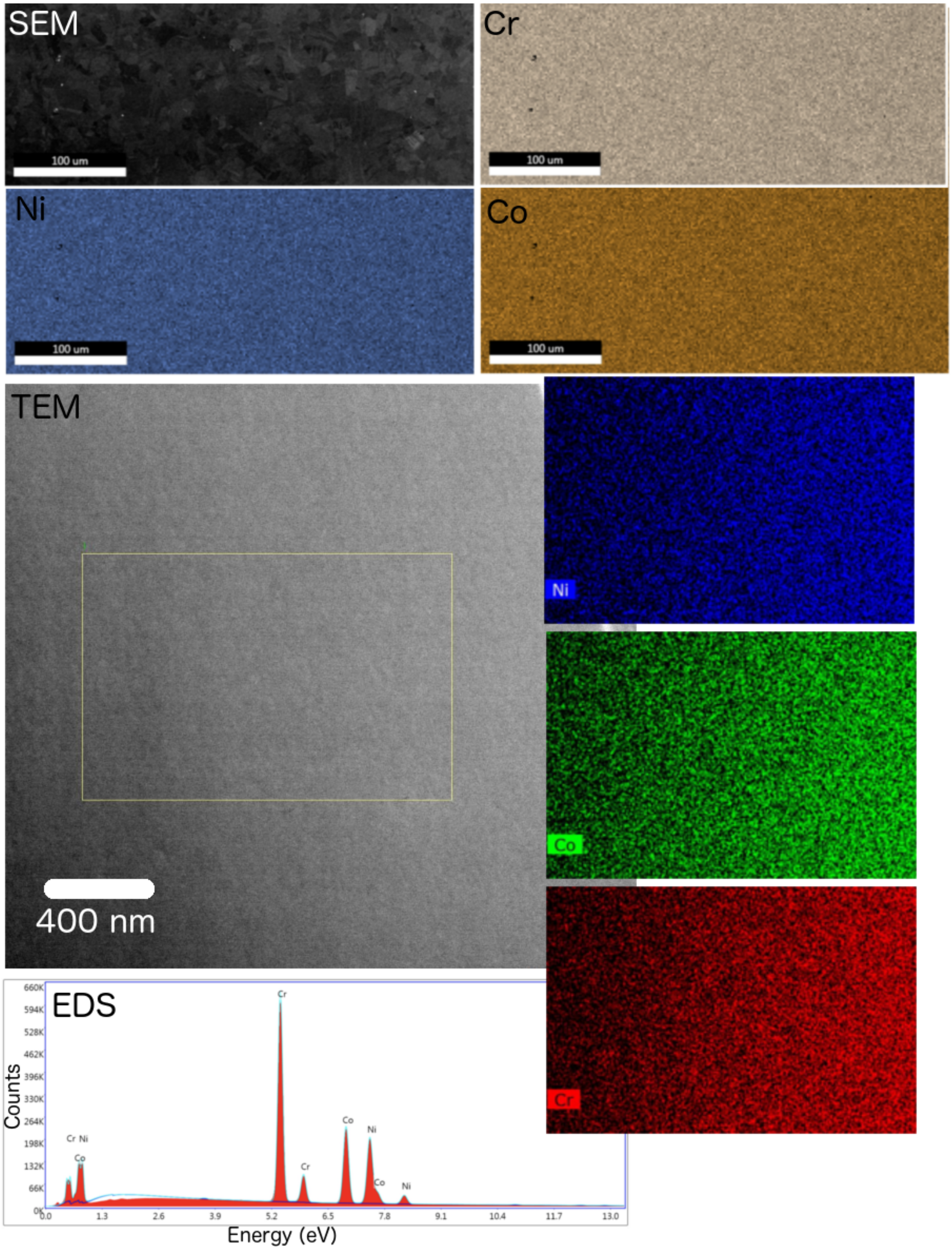

Fig. S1-2. Microstructure of annealed Alloy D ($Cr_{45}Co_{27.5}Ni_{27.5}$) observed by Scanning Electron Microscopy (SEM) and TEM, revealing compositional homogeneity and the single-phase FCC character of the alloy. The integrated energy dispersive spectroscopy spectrum of the alloy was collected in the SEM at an accelerating voltage of 20kV is also shown. The quantified spectra indicated 46.0 at% Cr, 27.1% Co, and 26.9% Ni, which are reasonable values considering the semi-quantitative nature of this technique.

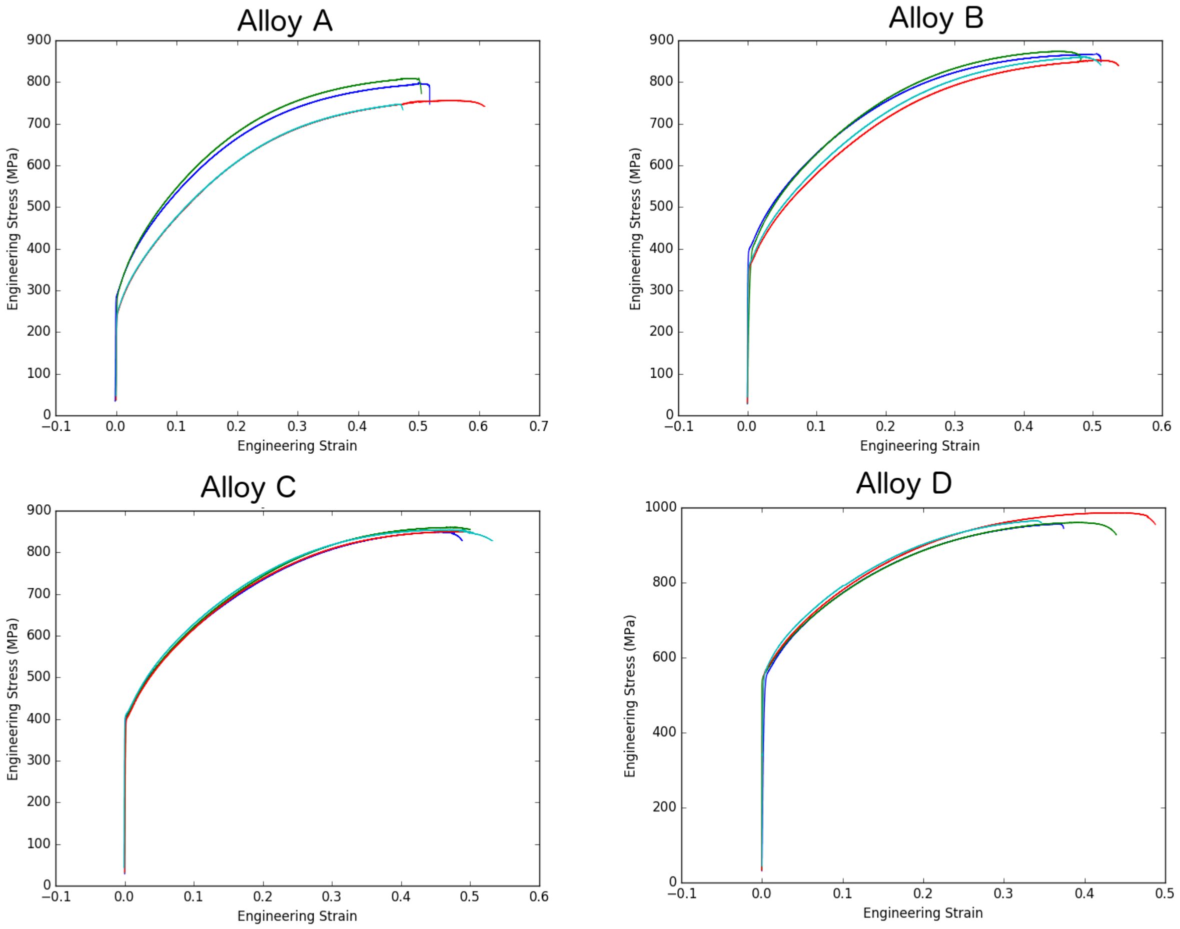

Fig. S1-3. Tensile engineering stress-strain curves of all the alloys A-D. The four samples from Alloys C and D had similar grain sizes and therefore each alloy had similar tensile behavior for all the tested samples. For Alloys A and B, some slight changes in the grain size between the two pairs of samples existed thus, more variation is seen. This grain size difference is likely due to different initial thicknesses and amounts of cold work reduction in the first pass.

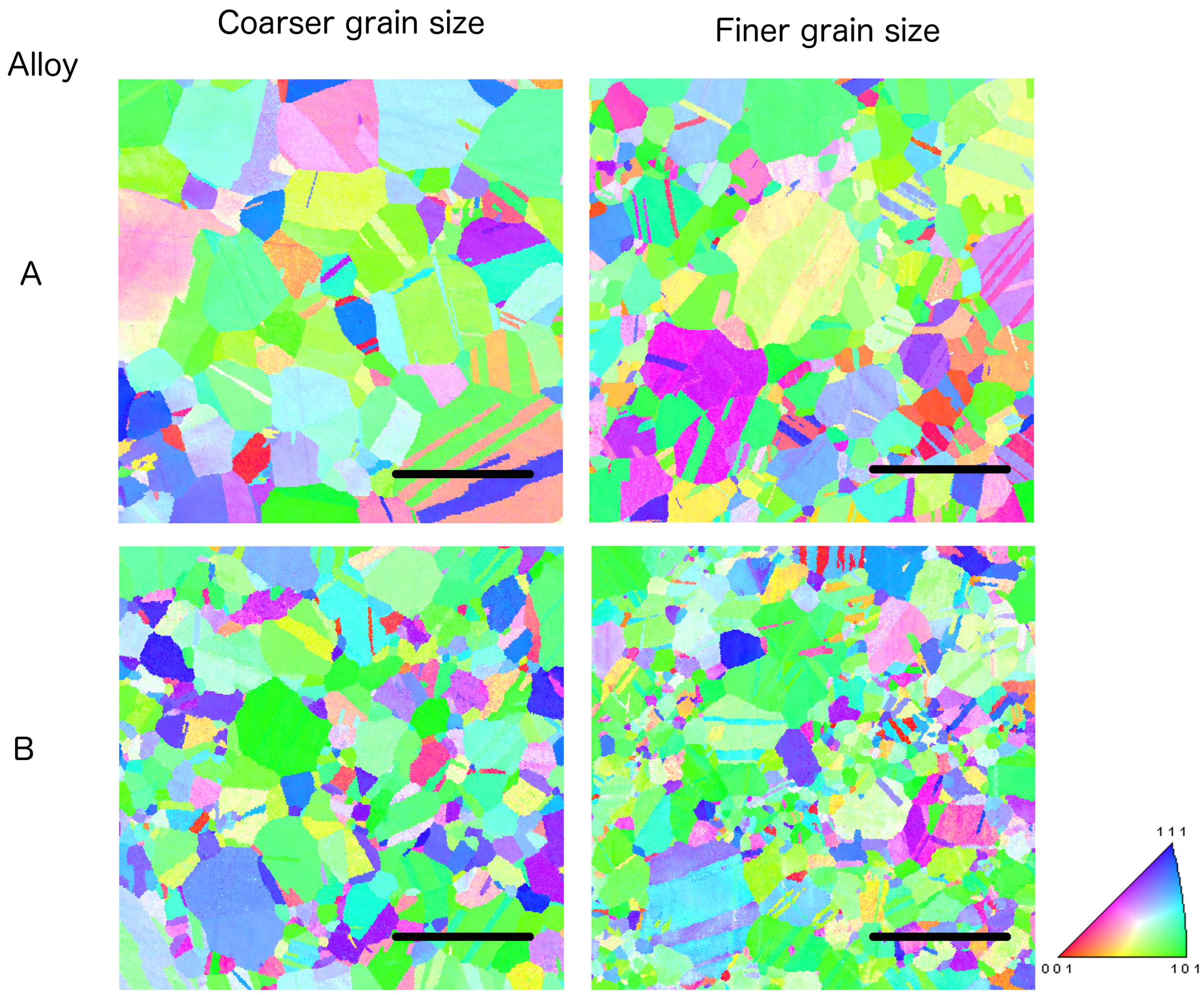

Fig. S1-4. Grain size differences between the two pairs of tensile samples of Alloys A and B, highlighted by EBSD inverse pole figure (IPF) maps. The conditions shown in Fig. 1 for Alloys A and B are those with finer and the coarser grain sizes, respectively, since these conditions better correspond to the grain size range of Alloys C and D. The scale bars in each IPF map correspond to 100 μm.

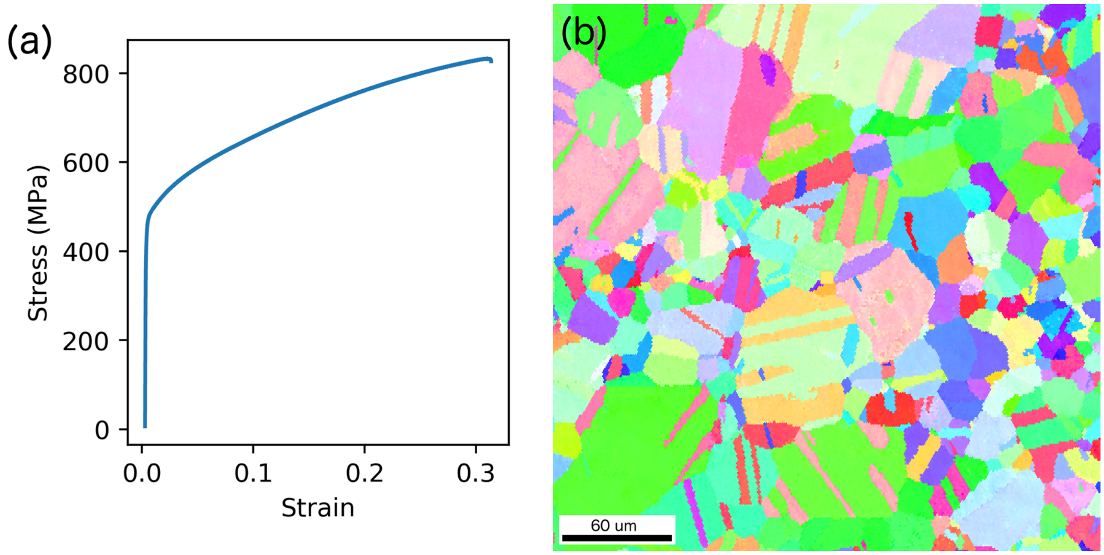

Fig. S1-5 (a) Tensile stress-strain curve and (b) IPF map obtained by EBSD of Alloy D heat-treated at 1130°C for 2 h. The yield strength is 455 MPa and the average grain size is around 80 μm.
